# Neuroprotective Effect of *Codonopsis pilosula* Polysaccharide on Aβ_25-35_-Induced Damage in PC12 Cells via the p38MAPK Signaling Pathways

**DOI:** 10.3390/ph17091231

**Published:** 2024-09-18

**Authors:** Liu Yang, Shiyi Song, Xinlu Li, Jinquan Wang, Yanan Bao, Xinxin Wang, Liwei Lian, Xiubo Liu, Wei Ma

**Affiliations:** 1 College of Pharmacy, Heilongjiang University of Chinese Medicine, Harbin 150040, China; yl13212980606@163.com (L.Y.); songshiyi2002@163.com (S.S.); 2 College of Jiamusi, Heilongjiang University of Chinese Medicine, Jiamusi 154007, China; l18747516492@126.com (X.L.); wsf19545316906@126.com (J.W.); byn13847642690@126.com (Y.B.); a1302196384@126.com (X.W.); llw123789456@126.com (L.L.)

**Keywords:** *Codonopsis pilosula* polysaccharide, Aβ_25-35_, PC12, damage, p38MAPK signaling pathway

## Abstract

Objectives: Plant polysaccharides have attracted increasing attention due to their high efficiency and low toxicity. *Codonopsis pilosula* polysaccharide (CPP) is an essential substance extracted from *Codonopsis pilosula*, known for its excellent antioxidant and neuroprotective effects. However, it is still unclear how CPP improves nerve protection and what its underlying molecular mechanisms are. This study aimed to investigate the neuroprotective effect of CPP on Aβ_25-35_-induced damage in PC12 cells and its underlying molecular mechanisms. Methods: The neuroprotective effect of CPP was evaluated using Aβ_25-35_-induced damage in pheochFfromocytoma (PC12) cells as an in vitro cell model. The cells were treated with CPP alone or in combination with SB203580 (an inhibitor of p38MAPK) in Aβ_25-35_ culture. The cell viability was assessed using a 3-(4,5-Dimethylthiazol-2-yl)-2,diphenyltetrazolium (MTT) assay. Furthermore, reactive oxygen species (ROS) were detected using flow cytometry. The production levels of intracellular superoxide dismutase (SOD), dismutase (SOD), glutathione (GSH), catalase (CAT), and malondialdehyFde (MDA) were determined using the colorimetric method. Annexin V-FITC and propidium iodide (PI) staining, as well as 33258 were performed using fluorescence microscopy. Moreover, the effect of adding SB203580 was studied to determine the changes in cell apoptosis induced by CPP treatment and Aβ_25-35_ induction. Results: The CPP markedly inhibited Aβ_25-35_-induced reduction in the viability and apoptosis of PC12 cells. CPP also reduced the Aβ_25-35-_induced increase in the expression of the apoptosis factors and the levels of free radicals (ROS and MDA) and reversed the Aβ_25-35_-induced suppression of antioxidant activity. Additionally, inhibition of p38MAPK via the addition of their antagonists reversed the observed anti-apoptosis effects of CPP. Conclusions: CPP can efficiently provide neuroprotection against Aβ_25-35_-induced damage in PC12 cells brought about via oxidation and apoptosis reactions, and the underlying mechanisms involve the p38MAPK pathways. Therefore, CPP could potentially be useful as a neuroprotective agent in natural medicine, pharmacy, and the food industry.

## 1. Introduction

Alzheimer’s disease (AD) is a neurodegenerative disease characterized by the deterioration of memory and cognitive behavioral abilities [1,2]. AD is mostly due to symptoms of near-memory impairment, and abnormalities in memory, recognition, expression, emotion, and personality gradually increase [3]. As the disease progresses, patients lose their individual and social abilities, experience a decline in the function of various organs, and develop various symptoms, ultimately leading to death from brain failure. AD is one of the most common types of senile dementia, affecting quality of life, compromising elderly health, and causing death in old age. As human social, technological, and medical sciences advance, human life expectancy increases, leading to a global aging population. Consequently, the incidence of AD is rising annually, with older patients facing a higher risk of developing the disease.

The underlying pathogenesis of Alzheimer’s disease (AD) involves diffuse brain atrophy. The main pathological features include senile plaques resulting from the accumulation and deposition of amyloid beta (Aβ) protein outside cells, neurofibrillary tangles resulting from the deposition of abnormally phosphorylated Tau protein inside cells, and widespread neuronal loss [4,5]. However, the pathogenesis of AD is complex and not entirely clear at present. In the quest for effective AD treatment, scholars from various countries have actively explored the pathogenesis of AD and proposed various hypotheses, such as neurotoxicity caused by Aβ deposition, abnormal phosphorylation of Tau protein, the cholinergic hypothesis, oxygenation stress response, and the cell apoptosis theory [6,7,8,9]. Oxidative stress and cell apoptosis have been the focus of recent research. Oxidative stress is a response that leads to an excess of reactive oxygen species in the body, resulting in the destruction of antioxidant enzymes [10,11]. The addition of oxygen free radicals to the neurotoxic process of AD promotes the deposition of Aβ and accelerates the process of cell apoptosis [12]. Neuronal apoptosis is a direct result of these mechanisms, leading to the loss of a significant number of neurons in the brain [13].

The apoptosis process is a complex process regulated by a variety of genes and enzymes. Among them, the Bcl-2 gene family inhibits apoptosis, while the Bax gene promotes apoptosis [14,15,16,17]. Additionally, oxidative stress can activate a series of signaling pathways in cells, leading to the irreversible halt of the cell cycle [18]. The P38MAPK pathway in the MAPK signaling cascade also plays a role in the apoptosis process of neurons by facilitating the translocation of Bax and activating caspase-3 [19]. Therefore, suppressing oxidative stress, cell apoptosis, and related signaling pathways may offer a potential approach to reduce nerve cell damage.

Since the pathogenesis of AD has not been fully clarified, clinical treatment of this disease is mainly aimed at improving the related clinical symptoms and delaying the progression of the disease. Commonly used drugs include cholinesterase inhibitors, NMDA receptor antagonists, non-steroidal anti-inflammatory drugs, nerve growth factors, and some drugs with antioxidant effects [20]. In addition, a large number of new drugs are also in the research stage [21,22]. The clinical efficacy of these drugs is certain, but the long-term benefits are still uncertain, and some drugs have obvious toxic side effects in long-term use. Therefore, it is urgent to explore effective and long-lasting therapeutic methods for treating AD with no toxic side effects.

*Codonopsis pilosula* (CP) is a well-known Chinese herb that has been used in traditional folk medicine for a long time in China, Japan, and Korea. CP is not only a food but also a medicine; it has the functions of invigorating qi, invigorating the spleen and lungs, nourishing blood, and promoting body fluid. Modern pharmacological studies have found that CP has functions such as anti-aging, anti-oxidative stress, and improving body immunity. *Codonopsis pilosula* polysaccharide (CPP) is the main active component of CP, which plays a neuroprotective role by improving neuronal damage and synaptic dysfunction caused by Aβ [23]. Some studies have reported that CPP can reduce the loss of synaptic plasticity in mice, increase synaptic protein, and play a certain neuroprotective role [24]. Additionally, CPP also alleviates cognitive impairment by reducing Tau phosphorylation levels in mouse models of Alzheimer’s disease [25]. However, whether CPP ameliorates AD pathological processes, especially oxidative stress, cell apoptosis, and related signaling pathways, has not been investigated yet, and further research is needed to answer this question.

In summary, we evaluated the neuroprotective effect of CPP in PC12 cells damaged by Aβ_25-35_. Subsequently, we used SB203580 to investigate the roles of p38MAPK-mediated pathways in the neuroprotective effect of CPP. This study offers a theoretical foundation for the development of CPP for the prevention or treatment of nerve impairment and broadens its application in natural medicine, pharmacy, and the food industry.

## 2. Results

### 2.1. Effect of CPP on Aβ_25-35_-Induced Cell Viability

**Figure 1:**
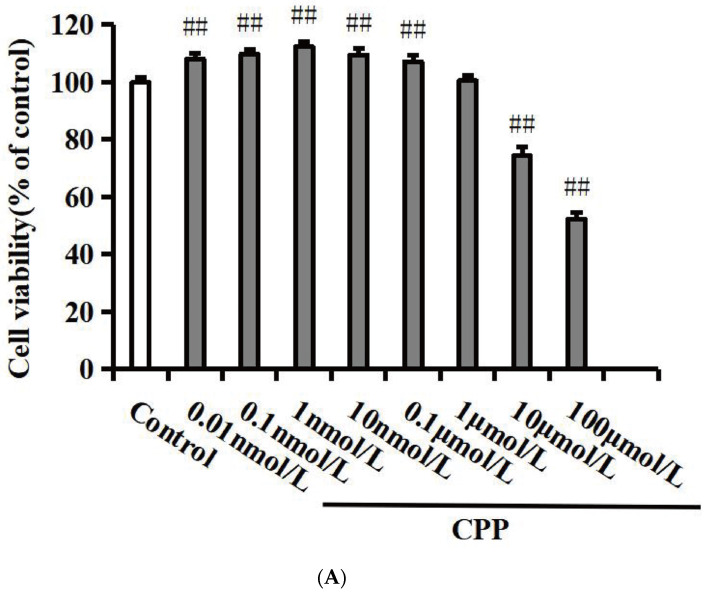

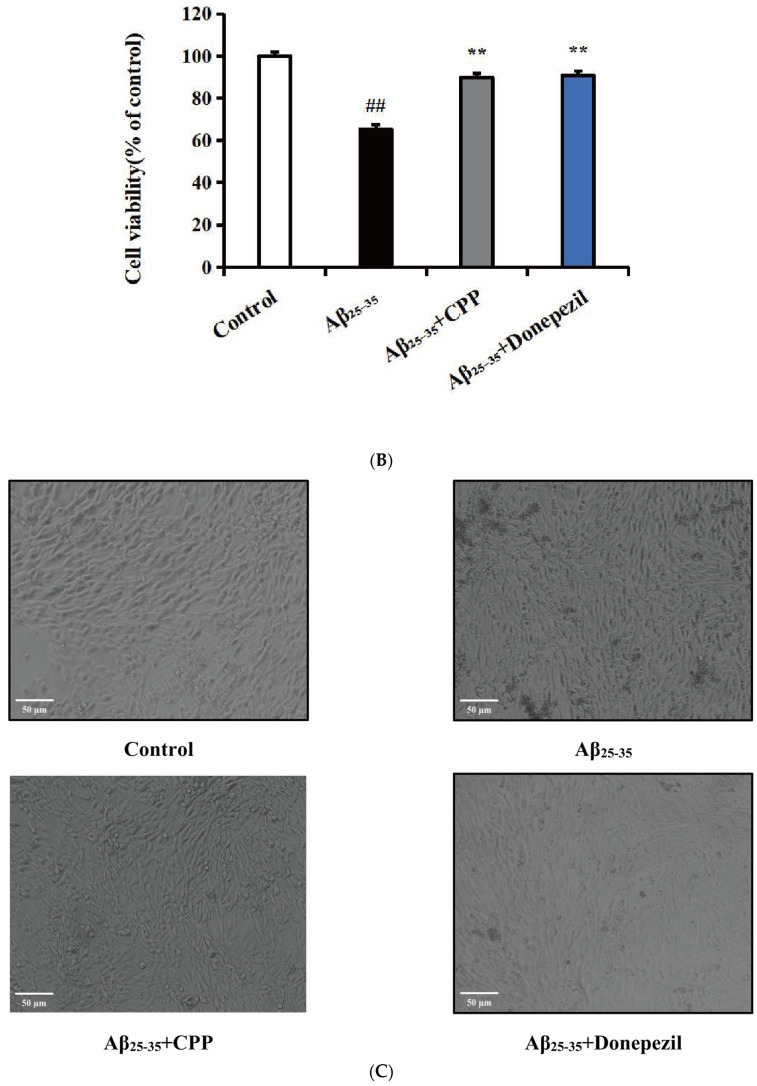
Effects of CPP on Aβ_25-35_-induced cell viability (n = 6). (**A**) Effect of CPP on cell viability. (**B**) Effect of CPP on Aβ_25-35_-induced cell viability. (**C**) Cell morphology observation. ^##^ *p* < 0.01 vs. the control group; ** *p* < 0.01 vs. the Aβ_25-35_ group.

To observe the possible protective effects of CPP, we evaluated PC12 cell viability at different concentrations. As shown in Figure 1A, CPP (0.01, 0.1, 1, 10 nmol/L, and 0.1 μmol/L) significantly promoted cell viability (*p* < 0.01) and CPP (10 and 100 μmol/L) showed significant inhibited cell activity and cytotoxicity (*p* < 0.01). When the cells were exposed to CPP (1 μmol/L) for 24 h, the cell viability demonstrated no significant change compared to the control group (*p* < 0.01). Therefore, CPP (1 μmol/L) was selected for subsequent experimental research.

In order to further verify the potential protective effects of CPP on Aβ_25-35_-induced damage in PC12 cells, we assessed PC12 cell viability in the presence of Aβ_25-35_, CPP, and donepezil at various concentrations. As depicted in Figure 1B, compared to the control group, the Aβ_25-35_ (20 μmol/L) group exhibited a significant inhibitory effect on cell activity (*p* < 0.01). Following treatment with CPP (1 μmol/L) and donepezil (10 μmol/L), the cell activity notably increased (*p* < 0.01). As illustrated in Figure 1C, the results demonstrated that the PC12 cells in the control group displayed intact morphology and structure, mostly fusiform with multiple synapses. In contrast, the model group exhibited significantly damaged cell morphology, enlarged cell spaces, cell fragments, reduced cell count, and diminished synapses. In comparison to the model group, the cell morphology in the CPP and donepezil group showed some improvement, with increased cell count, enhanced synapses, and more complete cell morphology. These findings suggest that CPP has protective effects on Aβ_25-35_-induced cell viability.

### 2.2. Effects of CPP on the Oxidative Stress in Aβ_25-35_-Induced Cells

**Figure 2:**
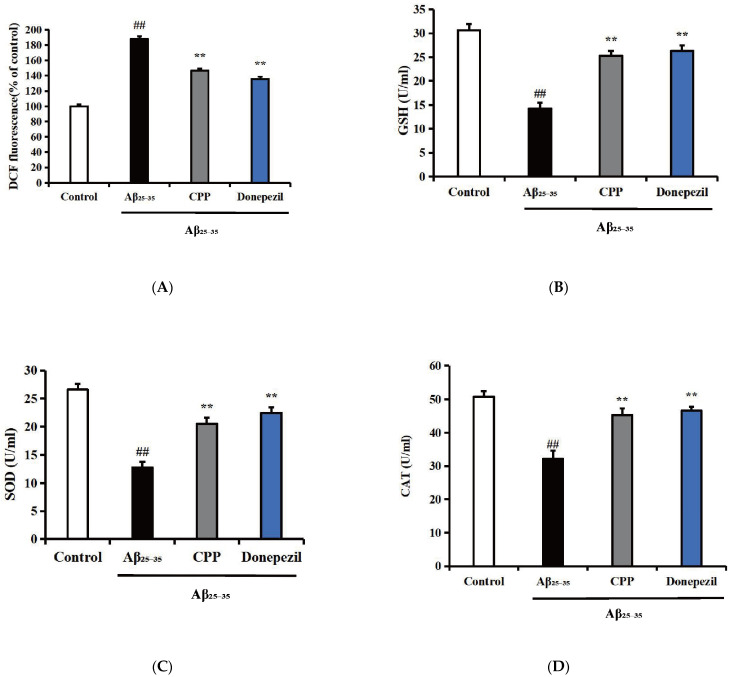

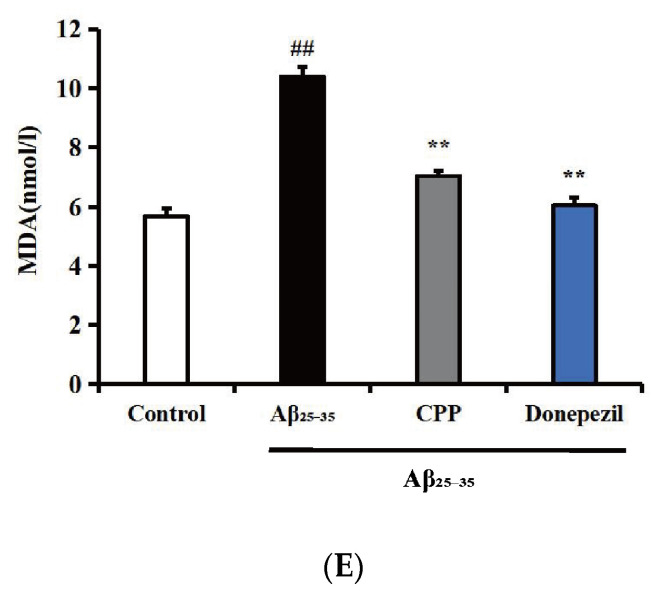
Effects of CPP on the oxidative stress in Aβ_25-35_-induced cells (n = 6). (**A**) Effects of CPP on the ROS in Aβ_25-35_-induced cells. (**B**) Effects of CPP on the GSH in Aβ_25-35_-induced cells. (**C**) Effects of CPP on the SOD in Aβ_25-35_-incuded cells. (**D**) Effects of CPP on the CAT in Aβ_25-35_-induced cells. (**E**) Effects of CPP on the MDA in Aβ_25-35_-induced cells. ^##^ *p* < 0.01 vs. the control group; ** *p* < 0.01 vs. the Aβ2_5-35_ group.

The intracellular levels of ROS, SOD, GSH, CAT, and MDA were determined to reflect the level of oxidative stress. As shown in Figure 2A,C, compared with the control group, the ROS and MDA levels in the Aβ_25-35_ group were significantly increased (*p* < 0.01). In comparison with the Aβ_25-35_ group, the ROS and MDA levels in the CPP and donepezil groups were decreased (*p* < 0.01). As shown in Figure 2B,D,E, compared with the control group, the GSH, SOD, and CAT levels in the Aβ_25-35_ group were significantly decreased (*p* < 0.01). In contrast, the GSH, SOD, and CAT levels in the CPP and donepezil groups were increased compared to the Aβ_25-35_ group (*p* < 0.01). These results indicate that CPP had an anti-oxidative stress effect in Aβ_25-35_-induced PC12 cells.

### 2.3. Effects of CPP on Apoptosis in Aβ_25-35_-Induced Cells

**Figure 3:**
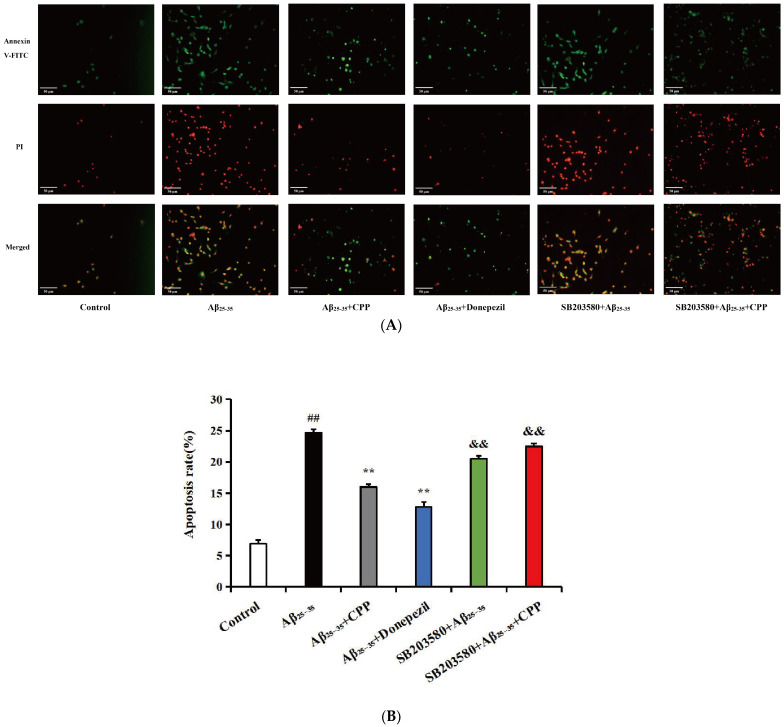

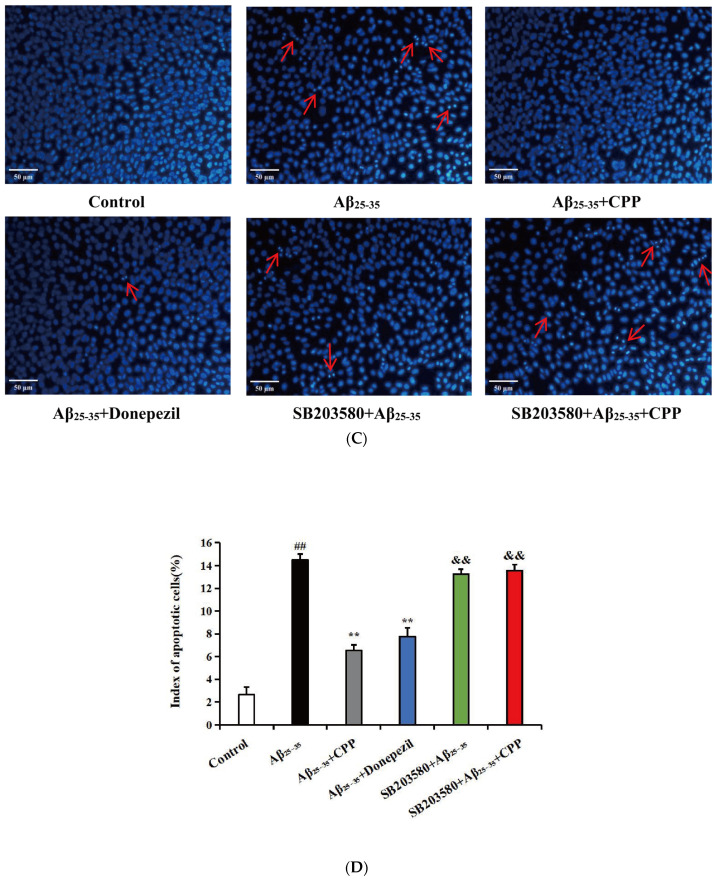
Effects of CPP on apoptosis in Aβ_25-35_-induced cells (n = 6). (**A**) Representative images by annexin V-FITC staining. The green fluorescence represents annexin V-FITC staining positive cells, while the red fluorescence indicates propidium iodide staining positive cells. Cells stained with green fluorescence were identified as apoptotic cells, those stained with red fluorescence were classified as necrotic cells, and cells not exhibiting any fluorescence staining were considered normal cells. (**B**) Apoptotic rate (%). (**C**) Representative images by Hoechst 33258 staining. The apoptotic bodies are indicated by arrows. (**D**) Index of apoptotic cells (%). The index of apoptotic cells (%) was determined by calculating the percentage of Hoechst positive cells over the total number of cells. ^##^ *p* < 0.01 vs. the control group; ** *p* < 0.01 vs. the Aβ_25-35_ group; ^&&^ *p* < 0.01 vs. the CPP group.

Cell apoptosis was analyzed using annexin V-FITC. As shown in Figure 3A, compared with the control group, the results indicate that the cells in the Aβ_25-35_ group exhibited more red and green fluorescence. The red and green fluorescence significantly decreased after the addition of CPP and donepezil drugs. However, upon adding SB203580, the red and green fluorescence significantly increased. In Figure 3B, compared with the control group, the results show that Aβ_25-35_ notably increased the rate of apoptosis (*p* < 0.01). The addition of the CPP and donepezil groups significantly reduced PC12 cell apoptosis compared to the Aβ_25-35_ group (*p* < 0.01). However, after adding SB203580, the apoptotic rate of the PC12 cells significantly increased (*p* < 0.01). These findings suggest that CPP may inhibit PC12 Aβ_25-35_-mediated cell apoptosis through the p38MAPK signaling pathways.

In addition, cell apoptosis was analyzed by Hoechst 33258 staining. As shown in Figure 3C, in the control group, the cell nuclei were normal, the fluorescence was light blue, and no apoptotic characteristics were observed. Compared with the control group, nucleus shrinkage and bright blue apoptotic bodies in the Aβ_25-35_ group were observed (*p* < 0.01). After CPP and donepezil intervention, the chromatin distribution was uniform and apoptotic bodies and nucleus shrinkage were significantly reduced, indicating that CPP could inhibit apoptosis induced by Aβ_25-35_ (*p* < 0.01). However, after adding SB203580, the nuclear shrinkage and bright blue apoptotic bodies were significantly increased (*p* < 0.01). As shown in Figure 3D, compared with the control group, the results show that Aβ_25-35_ markedly increased the index of apoptotic cells (*p* < 0.01). The addition of the CPP and donepezil groups markedly reduced PC12 cell apoptosis compared with that of the Aβ_25-35_ group (*p* < 0.01). However, after adding SB203580, the index of apoptotic cells was significantly increased (*p* < 0.01). This result also shows that CPP might restrain PC12 Aβ_25-35_-mediated cell apoptosis through the p38MAPK signaling pathways.

### 2.4. CPP Regulates mRNA Levels of Apoptotic Factors in Aβ_25-35_-Induced Cells through the p38MAPK Signaling Pathway

**Figure 4:**
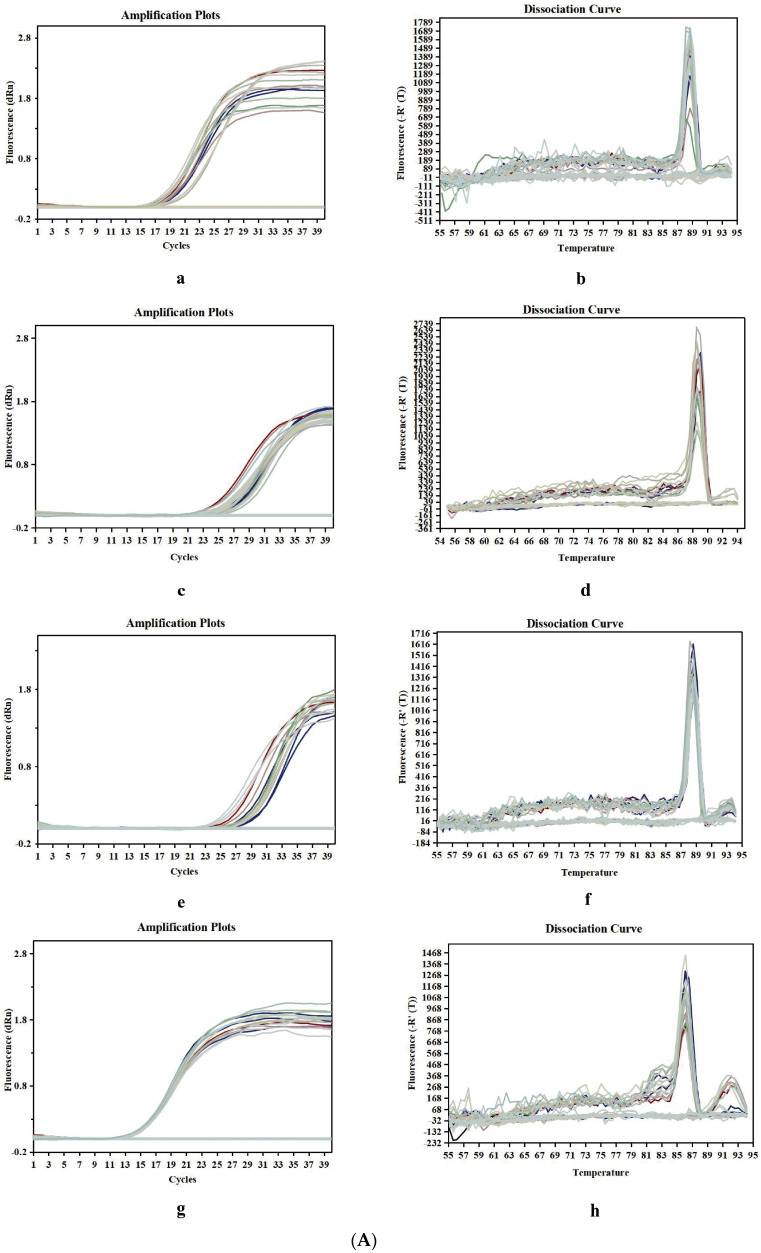

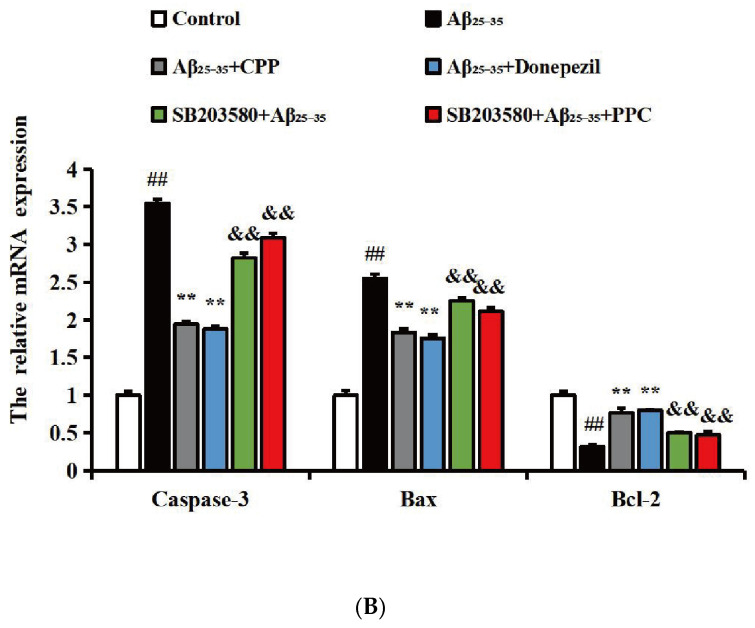
CPP regulates mRNA levels of apoptotic factor in Aβ_25-35_-induced cells through p38MAPK signaling pathway (n = 6). (**A**) Amplification and melting curves of caspase-3, Bax, Bcl-2, and β-actin. a—amplification curve of caspase-3, b—melting curve of caspase-3, c—amplification curve of Bax, d—melting curve of Bax, e—amplification curve of Bcl-2, f—melting curve of Bcl-2, g—amplification curve of β-actin, h—melting curve of β-actin. (**B**) Relative expression of mRNA. ^##^ *p* < 0.01 vs. the control group; ** *p* < 0.01 vs. the Aβ_25-35_ group; ^&&^ *p* < 0.01 vs. the CPP group.

To verify whether CPP exerts neuroprotective effects by regulating apoptosis factors associated with the p38MAPK signaling pathway, as shown in Figure 4A,B, compared with the control group, the results show that the expression of pro-apoptotic factors (Bax and caspase-3) was up-regulated and the anti-apoptotic factor (Bcl-2) was down-regulated in the Aβ_25-35_ group (*p* < 0.01). Compared with the Aβ_25-35_ group, CPP and donepezil significantly decreased the expression of the pro-apoptotic factors (Bax and caspase-3) and increased the expression of the anti-apoptotic factor (Bcl-2) (*p* < 0.01), whereas SB203580 significantly reversed these effects of CPP (*p* < 0.01). This result also shows that CPP played a neuroprotective role through the p38MAPK signaling pathway.

### 2.5. CPP Regulates Protein Levels of Apoptotic Factors in Aβ_25-35_-Induced Cells through the p38MAPK Signaling Pathway

**Figure 5:**
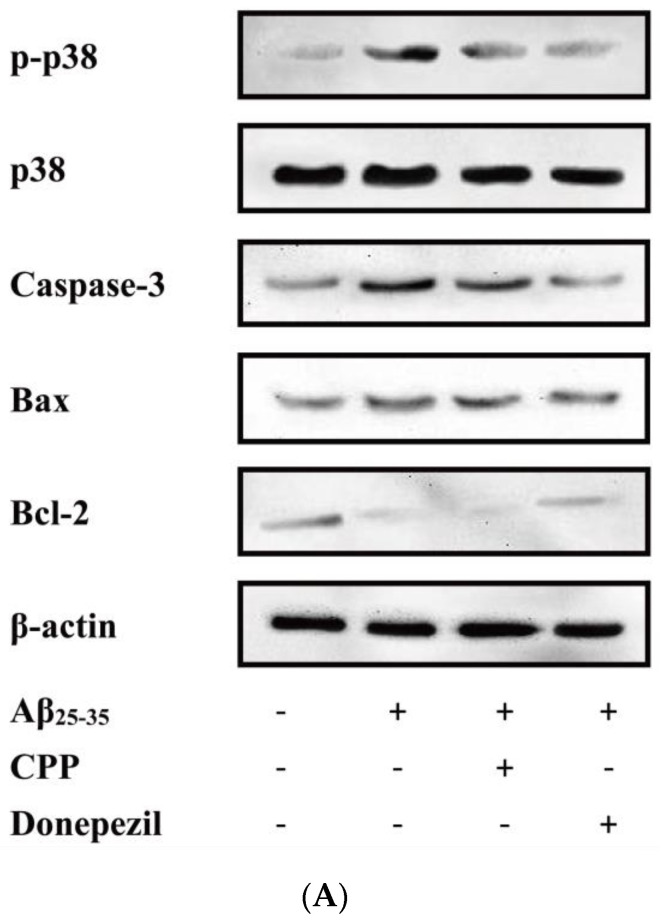

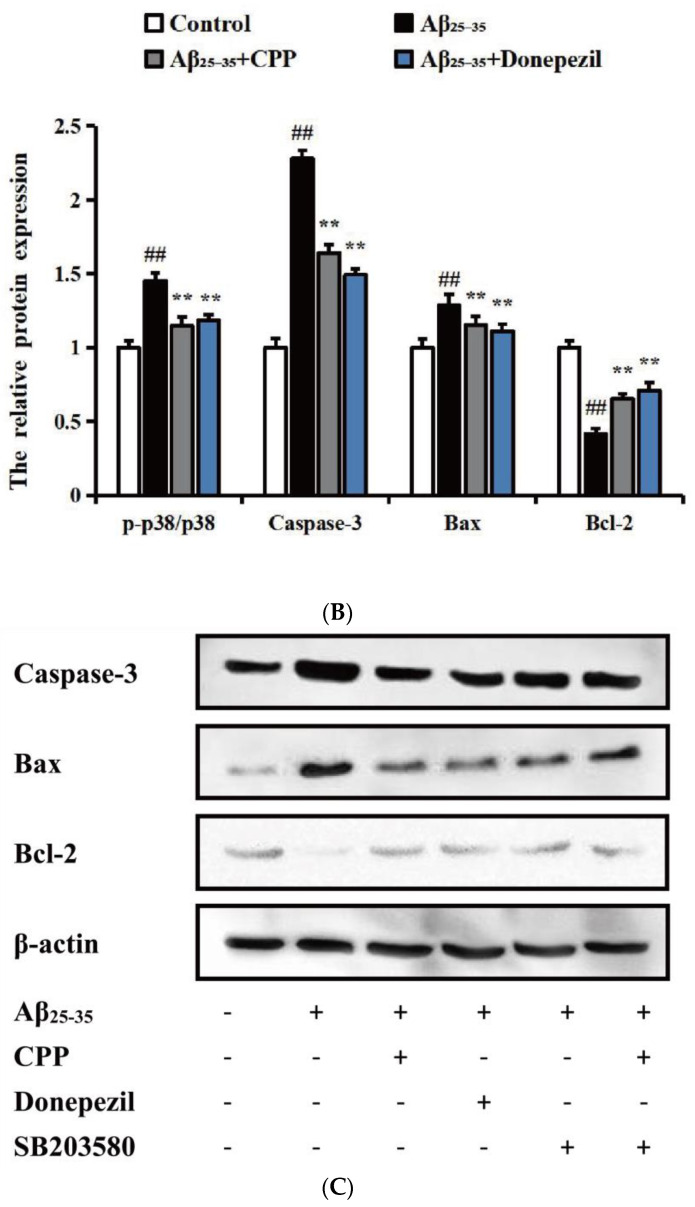

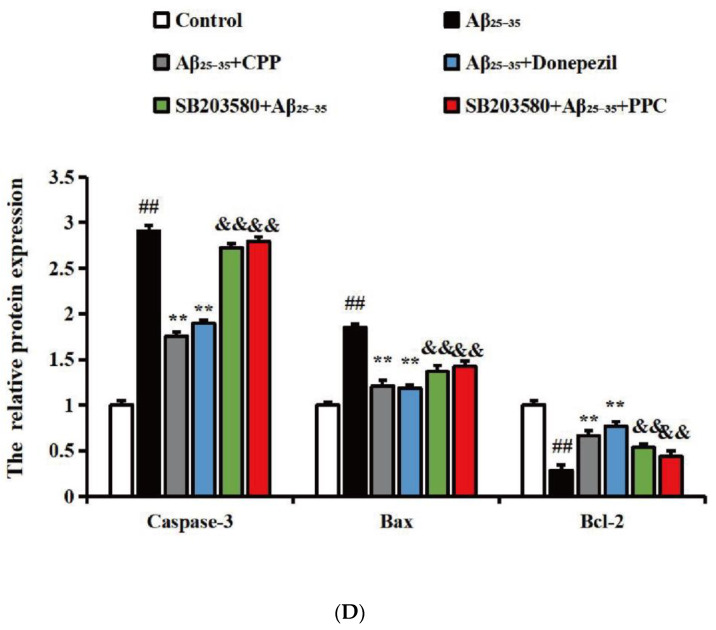
CPP regulates protein levels of apoptotic factor in Aβ_25-35_-induced cells through p38MAPK (n = 6). (**A**,**C**) Western blot images. (**B**,**D**) Relative expression of the protein. ^##^ *p* < 0.01 vs. the control group; ** *p* < 0.01 vs. the Aβ_25-35_ group; ^&&^ *p* < 0.01 vs. the CPP group.

To verify whether CPP exerts neuroprotective effects by regulating apoptosis factors associated with the p38MAPK signaling pathway, we measured the protein levels of apoptotic factors linked to p38MAPK. As shown in Figure 5A,B, compared with the control group, the results show that the expressions of Bax, caspase-3, p-p38, and p38 were up-regulated, while Bcl-2 was down-regulated in the Aβ_25-35_ group (*p* < 0.01). Compared with the Aβ_25-35_ group, CPP and donepezil significantly decreased the expression of Bax, caspase-3, p-p38, and p38 and increased the expression of Bcl-2 (*p* < 0.01). As shown in Figure 5C,D, with the addition of SB203580, compared with the CPP group, the results show that the expressions of Bax and caspase-3 were down-regulated, and Bcl-2 was up-regulated in the SB203580 group (*p* < 0.01). This result also indicates that CPP played a neuroprotective role through the p38MAPK signaling pathway.

## 3. Discussion

One of the main pathological manifestations of Alzheimer’s disease (AD) is the abnormal deposition of Aβ, with Aβ_25-35_ being the main toxic fragment. Aβ_25-35_ has been shown to damage PC12 cells, which closely resemble neurons. Apoptosis is a key outcome of Aβ-induced damage [26]. The rate of apoptosis in the brain nerve cells of AD patients is significantly higher than in normal individuals, which may be a crucial factor contributing to the decline of the central nervous system [27]. Aβ_25-35_ is a degradation product of the Aβ amyloid precursor protein (APP), a core component of amyloid plaques associated with aging. It accumulates in large quantities outside neurons, leading to neuronal loss or apoptosis through oxidative damage and other mechanisms. Following the induction of damage in PC12 cells by Aβ_25-35_, lipid peroxidation of polyunsaturated fatty acids on the cell membrane occurs, resulting in the generation of a substantial amount of myeloperoxidase. The stable metabolite MDA causes cross-linking and denaturation of phospholipids and proteins in cell membranes, releasing a significant number of stable cytoplasmic enzymes due to changes in membrane permeability [28]. GSH, SOD, and CAT are effective free radical scavengers in the human body, converting peroxides into harmless compounds. Experimental findings suggest that a considerable amount of reactive oxygen species are produced during the metabolic processes of AD. If the production and elimination of ROS are imbalanced, an accumulation of reactive oxygen species in the body can lead to oxidative stress damage [29].

Because of their high efficiency and low toxicity, plant polysaccharides have been gaining increasing attention [30]. Codonopsis polysaccharide (CPP) is a crucial active ingredient in Codonopsis, known for its anti-aging and immunity-boosting properties, as well as its ability to inhibit oxygen free radicals [31]. Studies have indicated that CPP can ameliorate learning and memory impairments induced by scopolamine and sodium nitrite and exhibit anti-aging effects in D-galactose-induced aging model mice. However, the potential of CPP to improve PC12 injury and oxidative stress induced by Aβ_25-35_ has not been explored. Therefore, in this study, PC12 cells were subjected to damage by Aβ_25-35_ to create an Alzheimer’s disease cell model. CPP was administered to the damaged cells, and its impact on the activity of ROS, GSH, SOD, CAT, and MDA was observed. Following CPP intervention, there was a significant increase in cell GSH, SOD, and CAT levels, while the ROS and MDA levels in the cells decreased significantly compared to the model group. This demonstrates that CPP can enhance the production of antioxidant enzymes in cells through its anti-oxidative properties, facilitating the removal of free radicals via catalytic disproportionation reactions, thereby reducing Aβ-induced cell apoptosis.

Aβ can cause an increase in pro-apoptotic factors and a decrease in anti-apoptotic factors through various pathways, ultimately leading to the degeneration, apoptosis, and necrosis of neurons [32]. Horiuchi M et al. [33] found that the overexpression and excessive deposition of amyloid beta protein are triggers of neuronal dysfunction, and the injection of Aβ into the brain can damage neurons. Therefore, antagonizing Aβ-induced apoptosis may be a treatment method for AD. The development of cell apoptosis is regulated by several genes and proteins, including the Bcl-2 family and the caspase family, which have been widely studied in previous AD research. Bcl-2 and Bax of the Bcl-2 family are mutually existing factors that inhibit and promote apoptosis in cytoplasmic death [34]. Bax and Bcl-2 proteins play crucial roles in the process of apoptosis [35]. Additionally, caspase-3 in the caspase family is a protease involved in the final execution of the cell apoptosis process [36,37]. Caspase-3 generally exists in a non-activated proenzyme state, which can trigger a protein kinase cascade reaction upon activation, leading to irreversible cell death [38,39].

**Figure 6:**
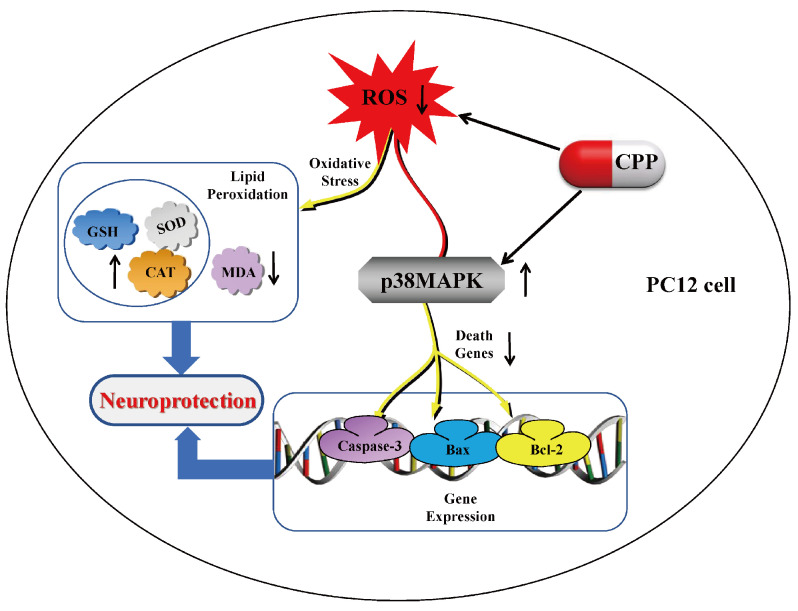
Neuroprotective effect of *Codonopsis pilosula* polysaccharide on A_β25-35_-induced damage in PC12 cells via the p38MAPK signaling pathways. In PC12 cells, CPP has a neuroprotective effect by reducing the content of reactive oxygen species, increasing the activity of antioxidant enzymes, and inhibiting the P38MAPK signaling pathway related to apoptosis.

Aβ oligomers are pathological components located outside neurons and must induce apoptosis through a series of signal transduction [40,41]. Mitogen-activated protein kinase (MAPK) is a classical signal transduction pathway that receives extracellular stimulus signals, amplifies and transmits them to the nucleus step by step, and participates in important cell events, such as cell proliferation, differentiation, and apoptosis [42]. p38MAPK is an important signaling pathway in MAPK that is mainly involved in the transmission of cellular inflammatory signals and cell apoptosis [43,44,45]. Many studies have revealed that p38MAPK is closely related to AD. Abnormal phosphorylation of p38MAPK can be found in the early stage of neurofibrillary deformation in the hippocampus of AD [46]. Moreover, previous studies have confirmed that the phosphorylation level of p38MAPK is positively correlated with the course of AD [47]. Yu et al. [48] showed that p38MAPK activation is involved in AD-mediated synaptic defects, oxidative stress, and mitochondrial dysfunction, and the use of drug-specific p38MAPK inhibitors can significantly increase synaptic density, inhibit oxidative stress, and improve mitochondrial dysfunction. Therefore, inhibition of p38MAPK phosphorylation may be a potential therapeutic strategy for the prevention and treatment of AD. Some studies have reported that the p38MAPK inhibitor (SB203580) can effectively regulate the apoptosis process of cells and the normal expression of related proteins and reduce the apoptosis of PC12 cells induced by Aβ_25-35_ [49]. The results of this study show that CPP could produce an anti-apoptotic pharmacological effect, which increased the expression of Bcl-2 and decreased the expression of Bax and caspase-3 in the model group. Under the action of SB203580, CPP reduced the expression of the Bcl-2 protein and increased the expression of p-p38/p38, Bax, and caspase-3 in the PC12 cells. These results suggest that the neuroprotective effect of CPP on A_β25-35_-induced damage in PC12 cells via the p38MAPK signaling pathways., as shown in Figure 6.

## 4. Materials and Methods

### 4.1. Materials

CPP and donepezil were acquired from Chengdu Must Biotechnology Co., Ltd. (Chengdu, China). Moreover, Dulbecco’s modified Eagle’s medium (DMEM), MTT, and dimethyl sulfoxide (DMSO) were purchased from HyClone (Logan, UT, USA). Fetal bovine serum (FBS) was acquired from Thermo Fisher Biochemistry Co., Ltd. (Beijing, China). All the antioxidant enzyme detection kits were obtained from Nanjing Jiancheng Biotechnology Co., Ltd. (Nanjing, China). Additionally, the BeyoECL Plus, annexin V-FITC kits, and Hoechst Staining Kit were provided by Beyotime (Shanghai, China). Furthermore, all the antibodies and SB203580 were purchased from Zhongshan Jinqiao Biotechnology Co., Ltd. and Bioss Biotechnology Co., Ltd. (Beijing, China).

### 4.2. Cell Culture and Experimental Design

PC12 cells were derived from Shanghai Zhongqiao Xinzhou Biotechnology Co., Ltd. (Shanghai, China). The cells were cultured in DMEM medium with 10% fetal bovine serum and 1% penicillin–streptomycin in a humidified atmosphere of 5% CO_2_ and 95% air at 37 °C. Initially, the 96-well plates were divided into different dose concentration groups, including the control group, CPP (0.01, 0.1, 1, 10 nmol/L and 0.1, 1, 10, 100 μmol/L). Based on previous experimental studies, it was established that a 20 μmol/L Aβ_25-35_ solution could damage cells by approximately 50% and was thus utilized in subsequent experiments [50]. To further investigate the protective effect of CPP on Aβ_25-35_-induced PC12 cells, the cells were divided into six equal groups: control group—no Aβ_25-35_ and CPP treatment; Aβ_25-35_ group—cells cultured in a new medium for 24 h, followed by incubation with Aβ_25-35_ (20 μmol/L) for another 12 h; CPP + Aβ_25-35_ group—cells incubated with CPP (1 μmol/L) for 24 h, then co-cultured with Aβ_25-35_ (20 μmol/L) for an additional 12 h; donepezil + Aβ_25-35_ group—cells incubated with donepezil (10 μmol/L) for 24 h, then co-incubated with Aβ_25-35_ (20 μmol/L) for another 12 h; SB203580 + Aβ_25-35_ group—cells incubated with SB203580 (10 mol/L) for 1 h, then cultured in a new medium for another 23 h, and finally co-cultured with Aβ_25-35_ (20 μmol/L) for an additional 12 h; SB203580 + CPP + Aβ_25-35_ group—cells incubated with SB203580 (10 mol/L) for 1 h, then incubated with CPP (1 μmol/L)for 23 h, and finally co-cultured with Aβ_25-35_ (20 μmol/L) for an additional 12 h.

**Figure 7:**
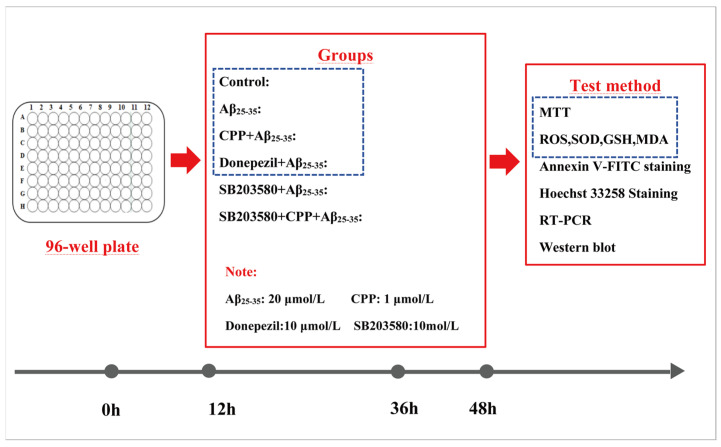
Cell experiment program. PC12 cells were divided into 6 groups for MTT, oxidative stress, apoptosis, Hoechst 33258 staining, RT-PCR, and Western blot analysis.

The experiments included the MTT assay; assessment with Hoechst 33258 staining; cell apoptosis assay; ROS, SOD, GSH, MDA production analysis; and RT-PCR and Western blot analysis under the experimental conditions (Figure 7).

### 4.3. Cell Viability Assay

The cells were incubated in 96-well plates. Each well was filled with 20 μL of MTT (5 g/mL) solution, incubated at 37 °C for 4 h, and then 200 μL of DMSO solution were added. The cell viability of each group was detected at 570 nm by a microplate reader (Shanghai, China). Meanwhile, the morphological changes of the PC12 cells in each group were observed under an inverted microscope (Tokyo, Japan). The cell viability rate (%) was calculated as follows: [cell viability rate (%) = OD570 administration group/OD570 control group × 100%].

### 4.4. Detection of Intracellular ROS Levels

The cells were collected and digested with trypsin for 2 min. After being cleaned three times with PBS, all the fluids were collected, and the cells were centrifuged at 1000 g for 5 min. After discarding the supernatant, DCFH-DA diluted in DMEM medium at a concentration of 1:1000 was added. The cells were then incubated in a cell incubator at 37 °C for 20 min and mixed upside down every 5 min to ensure full contact of the probe with the cells. Finally, the intracellular ROS levels were detected and recorded by flow cytometry (Hangzhou, China).

### 4.5. Detection of Intracellular SOD, GSH, CAT, and MDA Productions

After the cells were collected, they were washed three times with PBS, and RIPA lysis buffer was added. Subsequently, the cells were centrifuged at 12,000× g for 15 min at 4 °C. After centrifugation, the supernatant was analyzed according to the instructions provided with the kits.

### 4.6. Cell Apoptosis Detection

The cells were collected and centrifuged at 1000 g for 5 min. Subsequently, the supernatant was discarded, and the cells were resuspended in annexin V-FITC binding solution. The cells were then incubated with 5 μL of annexin V-FITC and 10 μL of PI staining solution for 15 min. Afterward, the cell suspension was dropped onto a slide, and the slide was covered. The annexin V-FITC fluorescence signal appeared green under blue excitation light, indicating apoptotic cells, while the PI fluorescence signal appeared red under green excitation light, indicating necrotic cells. Images were captured using a 10× objective lens. The cell data from 6 fields in each group were averaged (n = 6) and presented as the mean ± standard deviation ($\bar{x}$ ± s).

### 4.7. Nuclear Staining with Hoechst 33258

The Hoechst Staining Kit is a classic, fast, and convenient method for detecting apoptosis. When cells undergo apoptosis, chromatin shrinks. Therefore, after staining with Hoechst 33258, under fluorescence microscopy, the nucleus of normal cells appears blue, while the nucleus of apoptotic cells appears dense, densely stained, or fragmented and densely stained, and the color is somewhat white. After stimulating cell apoptosis, the culture medium was discarded, and 0.5 mL of fixing solution was added and fixed for 10 min. Then, the fixing solution was discarded, and the cells were washed twice with PBS for 3 min each time. The cells were then treated with 0.5 mL of Hoechst 33258 staining solution for 5 min and washed twice with phosphate-buffered saline (PBS). The cells were observed under fluorescence microscopy, the degree of nuclear aggregation/fragmentation was assessed, and the number of apoptotic cells was quantified. The formula for calculating the apoptosis index is as follows: (number of apoptotic cells in the treatment group/total number of cells in the treatment group)/(number of apoptotic cells in the control group/total number of cells in the control group).

### 4.8. RT-PCR Analysis

The total RNA was extracted using Trizol reagent, and the RNA content was measured by a Nano-100 microspectrophotometer (Hangzhou, China) (Table 1). Subsequently, the RNA was reverse transcribed into cDNA using a kit. The cDNA was used for an SGExcel UltraSYBR mixture. The reaction conditions were as follows: 1 cycle at 95 °C for 10 min, 40 cycles at 95 °C for 30 s, 55 °C for 60 s, seconds, and 72 °C for 60 s. Real-time quantitative PCR (Beijing, China) was used for detection. The relative expression of the target gene was calculated by a 2^−∆∆Ct^ value. The primer sequence is shown in Table 2.

### 4.9. Western Blot Analysis

Firstly, the collected cells were fully lysed with RIPA cell lysis buffer, and the total protein concentration was determined using the BCA method. Subsequently, equal amounts of the samples were separated by 10% sodium dodecyl sulfate polyacrylamide gel electrophoresis (SDS-PAGE), and the proteins were transferred to PVDF membranes. The membranes were then blocked with a 5% sealing solution for 2 h and incubated with the primary antibody overnight at 4 °C. Following this, the secondary antibodies were incubated for 2 h. β-actin served as the reference protein. After washing the membranes, an enhanced chemiluminescence (ECL) solution was applied for detection, and the gray values were analyzed using a gel analysis system (Smart Chemi II, Saizhi Venture, Beijing, China).

### 4.10. Statistical Analysis

All the statistical data were expressed as mean ± standard deviation (SD) and processed using the SPSS 18.0 software package. Based on the test results for homogeneity of variance, one-way analysis of variance (ANOVA) was employed for statistical analysis among multiple groups, with values of *p* < 0.05 or *p* < 0.01 considered statistically significant.

## 5. Conclusions

In conclusion, this study demonstrates the neuroprotective effect of Codonopsis pilosula polysaccharide on Aβ_25-35_-induced damage in PC12 cells through the regulation of oxidative stress and cell apoptosis. The underlying mechanisms involve the p38MAPK pathways. Thus, CPP could potentially be useful as a neuroprotective agent in therapeutic and/or dietary healthcare products.

## Figures and Tables

**Table 1:** The purity and concentration of RNA were detected using an ultraviolet light.

Groups	Concentration (ng/μL)	Absorbance Value OD (260/280)
Control	282.181 ± 6.321	1.9221 ± 0.0241
Aβ_25-35_	240.011 ± 3.549 ^##^	1.8687 ± 0.0403 ^##^
Aβ_25-35_ + CPP	261.056 ± 4.543 **	1.8920 ± 0.0124 **
Aβ_25-35_ + donepezil	262.326 ± 3.432 **	1.9032 ± 0.0333 **
SB203580 + Aβ_25-35_	244.153 ± 3.654 ^&&^	1.8942 ± 0.0264 ^&&^
SB203580 + Aβ_25-35_ + CPP	253.246 ± 7.422 ^&&^	1.9824 ± 0.0502 ^&&^

^##^ *p* < 0.01 vs. the control group; ** *p* < 0.01 vs. the Aβ_25-35_ group; ^&&^ *p* < 0.01 vs. the CPP group.

**Table 2:** Gene-specific primers used for the RT-PCR.

Primer Name	Primer Sequence—Forward (5′–3′)	Primer Sequence—Reverse (5′–3′)	Length (bp)
β-actin	GACTTAGTTGCGTTACACCCTTTC	GCTGTCACCTTCACCGTTCC	160
Caspase-3	CCAAAGATCATACATGGAAGCG	CTGAATGTTTCCCTGAGGTTTG	185
Bax	CGAACTGGACAGTAACATGGAG	CAGTTTGCTGGCAAAGTAGAAA	157
Bcl-2	GACTTCGCCGAGATGTCCAG	GAACTCAAAGAAGGCCACAATC	129

## Data Availability

The original contributions presented in this study are included in the article. Further inquiries can be directed to the corresponding authors.
